# Feasibility of an Extensive Strategy for Adult Diagnosis of Attention Deficit Hyperactivity Disorder Among Patients Suffering From Substance Use Disorders

**DOI:** 10.3389/fpsyt.2022.803227

**Published:** 2022-06-28

**Authors:** Norman Therribout, Emily Karsinti, Alix Morel, Alexandra Dereux, Florence Vorspan, Lucia Romo, Romain Icick

**Affiliations:** ^1^Assistance Publique – Hôpitaux de Paris, Hôpital Fernand-Widal, Département Universitaire de Psychiatrie et de Médecine Addictologique, Paris, France; ^2^Université de Paris Cité, INSERM UMR-S1144, Paris, France; ^3^Laboratoire Clipsyd EA 4430, Université Paris-Nanterre, Nanterre, France; ^4^FHU NOR-SUD Network of Research in Substance Use Disorders, Paris, France; ^5^Assistance Publique – Hôpitaux de Paris, Hôpital Raymond-Poincaré, Garches, France; ^6^CESP, U1018 INSERM UPS UVSQ, Villejuif, France

**Keywords:** diagnosis, acceptability, stimulant, cognitive, cocaine, attention deficit-hyperactivity disorder (ADHD), dual disorder (DD), substance use disorder (SUD)

## Abstract

**Introduction:**

Attention Deficit Hyperactivity Disorder (ADHD) is found in up to 20% adults with Substance Use Disorder (SUD). ADHD + SUD is associated with a more complex clinical presentation and poorer outcomes than each disorder alone. In the presence of SUD, adult ADHD is particularly difficult to diagnose as both disorders can mimic or hide the symptoms of each other. Our university hospital in Paris recently started an extensive outpatient diagnostic procedure for adult patients with SUD to ascertain or refute ADHD diagnosis and to provide therapeutic guidance. Here, we report the acceptability of the assessment procedure for patients and the preliminary description of the current and lifetime clinical profiles as a function of the final diagnosis “ADHD vs. no ADHD.”

**Method:**

Adult SUD patients with suspected ADHD were included in the current pilot study after stating they had no objection that their de-identified data were used for research purposes, according to French ethical procedures. Patients were evaluated for ADHD, comorbid mental disorders, cognitive state and dimensional psychological variables. They were assessed by trained psychologists and psychiatrists using standardized tools over a day. ADHD diagnosis was mainly based on the Diagnostisch Interview Voor ADHD for DSM-5 (DIVA-5).

**Results:**

Out of 18 eligible patients, 17 were included in the cohort (1 excluded) and none was opposed to using their data. Thirteen (76%) participants were diagnosed with ADHD. All patients appointed for the ADHD diagnostic procedure came, respected schedules and finished the evaluation. All patients were impaired on cognitive functioning and were highly comorbid, but ADHD patients seems to suffer even more from those conditions, especially for cannabis and stimulant use disorders.

**Discussion:**

Preliminary results show high acceptability of the procedure by ADHD-SUD patients. This result could be explained by all the organization adapted to the psychopathology. Patients' baseline motivation to participate also represents an uncontrolled variable that could promote the ability to follow the procedure. Acceptance results of the protocol are promising and represent a starting point to identify the best procedures to design patient-centered pharmacological and non-pharmacological therapies.

## Introduction

Attention Deficit Hyperactivity Disorder (ADHD) is a pervasive neurodevelopmental disorder that is likely to persist into adulthood (1). In the general population, ADHD is found in 2.6% adults. This prevalence raises up to 6.8% when the presence of ADHD during childhood—a prerequisite for adult diagnosis according to several classification systems—is not considered (2). One of the most burdensome comorbidity of ADHD is substance use disorder (SUD), which often develops when ADHD persists throughout adolescence, so that up to 20% patients seeking treatment for SUD suffer from comorbid ADHD (3). Prevalence of ADHD in SUD adults varies across culture, substance and methodologies, from 2% in Islandic adolescents (4) to 83% in Japanese stimulant abusers (5). Standardized clinical interview in methodology instead of questionnaires resulted in a prevalence variability reduction at 5.4–34.3% (6), emphasizing the need of clinical interview, especially to take into account socio-cultural aspects (7).

Comorbid ADHD is associated with more severe patterns of SUD (8), including higher rate of poly-dependence, earlier onset (9), and cocaine-induced psychotic symptoms (10). Consequently, diagnosing ADHD in people with SUDs is of utmost importance.

The overlapping symptoms between ADHD and SUD represent a challenge for ADHD diagnosis procedure and treatment (11). Both disorders seem to have a bi-directional causal relationship with common symptoms contributing to maintain both disorders (12). Several instruments allow for screening and diagnosing ADHD in adult populations, however, they present limitations when used in SUD population, especially if used in an isolated manner. Regarding screening tools, the six-item World Health Organization's Adult ADHD Self-Report Scale (ASRS-6) has been validated in SUD populations (13), however, the ASRS still yields high rate of false negatives in SUD population (14). This has also been observed with the Conner's ADHD Adult Rating Scale (CAARS). The Wender Utah Rating Scale (WURS) may be a relevant complementary strategy to increase the screening accuracy of ADHD in this population (15, 16).

As for diagnostic tools, the Conner's ADHD Adult Diagnostic Interview for DSM-IV (CAADID) is often considered as the golden standard to diagnose ADHD, including in SUD adult population. Unfortunately, it remains only available in English and Dutch and, while providing in-depth investigations such as age at onset of each ADHD symptom, it remains mostly based on DSM-IV classification and its length can be a downside in SUD populations. When compared with the CAADID, the ADHD section of the Psychiatric Research interview for Substance and Mental Disorders (PRISM) showed good psychometric properties to detect ADHD in SUD population (17), yet again being based on DSM-IV criteria. The ADHD module of the Mini International Neuropsychiatric Interview (MINI) showed promising criterion validity in treatment-seeking SUD patients (18). Finally, the *Diagnostisch Interview Voor ADHD* (DIVA-5) has recently been translated in French and allows for both child and adult ADHD diagnosis while assessing functional impairment. The first validation study of the DIVA-5 concluded that it seemed to be a reliable tool in a Korean population (19). Overall, several screening and diagnostic instruments for ADHD have been developed, but most of them remain only available for specific languages and/or former DSM versions.

In this context of unmet diagnostic needs for ADHD, neurocognitive measures may hold promises in the ADHD diagnosis procedure, particularly regarding processing speed and working memory (20). However, the cognitive profile is easily affected by the presence of comorbidities such as depressive disorder (21) and should only be considered as a support for the diagnostic procedure (22, 23).

Available evidence highlighted that assessing ADHD among SUD population is profitable to both the diseases (24, 25). The international consensus on screening, diagnosis and treatment of SUD with comorbid ADHD (26) thus recommends a systematic screening of ADHD in SUD populations and *vice-versa*.

In order to address the major issue of diagnosing ADHD in SUD individuals, we developed an extensive assessment procedure that occurs over a day in our public academic hospital. Our main research aims are to validate the French versions of several diagnostic instruments in their DSM-5 versions and to detect potential neurocognitive profiles of ADHD in this population. For the current report, we chose to provide the preliminary descriptive and comparative statistics of the first case series of included patients. We focused on participants' ability to undergo the full procedure and on the SUD and main cognitive characteristics of those eventually diagnosed with ADHD vs. those who were not. Our hypotheses were that at least 5% participants would have serious difficulties in fulfilling all assessments and that ADHD would show clinical profiles suggestive of increased severity.

## Materials and Methods

### Participants

Participants were unpaid adult French-speaking outpatients receiving medical or psychological care for SUD. Fifty three percent presented a severe SUD pattern and 24% were in early remission. Recorded by the Weiss Functional Impairment Rating Scale self-report (WFIRS), the largest functional impairment is reported in self-concept, followed by school field and life skills. Inclusion criteria for the current study were the same as the expert assessment that is conducted at our day hospital for addiction medicine. Patients were referred from primary or tertiary addiction care settings by word-of-mouth to ascertain or refute adult ADHD diagnosis. They underwent a full diagnostic procedure, whichever their comorbidities, provided that they fulfilled a set of screening questionnaires, including: a free text form summarizing the referral's motives for assessment and participants' current treatment and medical history; Adult ADHD Self-Report Scale 6 items version for DSM-IV (ASRS-6, the DSM-5 version being unavailable at the time of the current study); the Wender Utah Rating Scale, 25-items (WURS-25), the Alcohol Use Disorder Identification Test (AUDIT); the Cannabis Use Disorder Identification Test (CUDIT) and the Fagerström Test for Nicotine Dependence (FTND).

There was no additional inclusion criteria for the current study. Additional exclusion criteria were: unable to complete assessments due to unstable medical condition (including acute intoxication), compulsory admission, or current guardianship. According to the French ethical bylaws, patients could be included without signing written informed consent, if they did not express their opposition to participate and that their data were pseudonymized. The study was conducted according to the tenets of the Declaration of Helsinki (Declaration of Helsinki, 2013) and of Paris-Nanterre University ethics committee rules (CPP sud-est IV, on February 22, 2021).

### Assessments

Before assessments, all participants were contacted by phone to properly describe the whole assessment procedure and to arrange an appointment, which was further confirmed by phone text-message. Once they arrived on site, they were also accompanied by a nurse to carry out administrative procedures and to collect vital signs (heart rate, blood pressure, weight, urine drug screening, and alcohol breath-testing). This moment also allowed flexibility for late arrivals.

**Figure 1:**
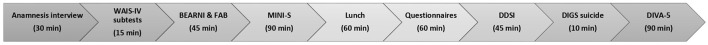
Evaluation procedure. WAIS-IV, Wechsler Adult Intelligence Scale fourth edition; BEARNI, Brief Evaluation of Alcohol-Related Neuropsychological Impairment; FAB, Frontal Assessment Battery; MINI-S, Mini International Neuropsychiatric Interview DSM-5 edition; DDSI, Dual Diagnosis Screening Instrument; DIGS, Diagnostic Interview for Genetic Studies, suicide module; DIVA-5, Diagnostic Interview for ADHD DSM-5 edition.

All assessments were conducted face-to-face with trained psychiatrists and psychologists, who were assigned different questionnaires between participants. A typical assessment day includes (Figure 1):

- An anamnestic interview for the main clinical and socio-demographic background, including the number of DSM-5 criteria for the main current substance use;
- We assessed a range of cognitive functions in two steps, using:

- three subtests of the Wechsler Adult Intelligence Scale (WAIS-IV) encompassing Processing Speed and Working Memory (Symbols, Code, and Number Memory subtests). The WAIS (27) is the most commonly used battery to assess intellectual functioning (28), and impairments in both processing speed and working memory have been identified in adults with ADHD (20);
- a screening of cognitive dysfunction with the Brief Evaluation for Alcohol Related Neuropsychological Impairment (BEARNI) and the Frontal Assessment Battery (FAB).

- Psychiatric and addictive comorbidities were then ascertained using the Mini International Neuropsychiatric Interview Simplified for DSM-5 (MINI-S). The MINI-S provides categorical diagnoses for 13 psychiatric disorders, including SUDs, and their current remission status. To date, the Mini-S had been validated for the depressive symptoms (29). However, the MINI-Plus based on DSM-IV criteria has shown acceptable validity for the screening of adult ADHD in SUD samples (18). Participants also underwent Dual Disorder Screening Instrument (DDSI), as part of the primary cohort objective of French validation (30).
- Lifetime history of suicidal attempt was collected using the “suicide” section of the Diagnostic Interview for Genetic Studies, v 4.0 (DIGS 4.0) (31). The first questions on the presence (and number) of lifetime suicide attempts were followed by an assessment of the self-reported worst attempt (method, intention to die).
- Participants were then offered lunch onsite for 60 min. Afterwards, they were asked to complete four self-rating scales aimed to estimate (i) the functional impact of their symptoms using the Behavior Rating Inventory of Executive Function Adult version – BRIEF-A (32) and the Weiss Functional Impairment Rating Scale self-report – WFIRS) (33), (ii) anxiety and depression levels (Hospital Anxiety and Depression scale – HAD) (34, 35) and (iii) trait-impulsiveness (Urgency, Premeditation, Perseverance, Sensation Seeking, Positive Urgency, Impulsive Behavior Scale – UPPS-P) (36).
- Finally, all participants underwent the Diagnostic Interview for ADHD in adults (DIVA-5) to investigate ADHD symptoms during childhood and adulthood and ascertain the diagnosis, regardless from the results on ADHD screening scales. Importantly, this questionnaire allows collecting hetero-anamnestic data from child health record, parents' testimony, teachers' evaluations and comments on academic transcripts to reinforce diagnostic reliability. The DIVA is one of the structured interviews recommended in adults with SUDs, for whom ADHD is suspected by clinicians, whether the screening was positive or not (26).

The time allowed for each assessment (indicated in brackets on Figure 1) was higher than the time typically required to permit regular breaks during the day and increase the overall flexibility of the assessment procedure. We identified a high heterogeneity in assessments durations, particularly for semi-directive diagnostic interviews. We explain longer evaluations in two ways: patients' difficulties to focus on the one hand, and the presence of psychiatric comorbidities, requiring specific symptoms investigations on the other hand. Overall, patients stayed at the unit form 8:30 a.m. to 4:30 p.m. The range of the tools assessing each domain of interest remained relatively restricted. This was deemed *a priori* in order to maintain a good balance between collecting data relevant for the clinics and research and yielding a feasible assessment procedure.

A second appointment was proposed to each participant for a debriefing session during which the final diagnosis and therapeutic guidance were discussed, along with basic psychoeducation regarding ADHD and/or comorbid disorders.

### Statistical Analyses

For the current descriptive study, we report the preliminary results from anamnestic self-reports regarding sociodemographic data and current substance use, the DIVA-5, the MINI-S, the BEARNI and the FAB. First, descriptive statistics were calculated to examine characteristics of the total sample. Second, these clinical and sociodemographic variables were described as a function of the presence/absence of current ADHD according to the DIVA-5. Data were roughly classified into sociodemographics, SUD, mental disorders other than SUDs and ADHD, and ADHD data—if applicable. Third, we selected the most salient descriptive results to plot relevant data, according to these categories. We used R and Rstudio on Mac OS X.12.3.

## Results

### Preliminary Data About Feasibility

All participants (*n* = 18) attended, respected their schedule and attended the entire evaluation procedure. One participant showed external signs of discomfort and irritability. The others reported good subjective tolerance to the procedure. They pointed out to the protocol length and reported subjective tiredness but found it bearable due to previous notice regarding the evaluation procedure and internal motivation to investigate their symptomatology. One of these, however, was excluded of the protocol because of unstable medical condition, leaving a study sample of 17 participants.

### Total Sample

Sample characteristics are described in Table 1. Participants were 37 years old (interquartile range, IQR = 29–41), 10 (59%) were men, three (18%) were in a relationship, thirteen (76%) participants had a high school degree or higher and ten (59%) were currently unemployed (including one retired person and one on disability leave). Current substance use was as follows: ten (59%) tobacco smokers, fifteen (88%) alcohol users, eight (47%) cannabis smokers, five (29%) cocaine users, and two (12%) opioid users. Main SUD diagnosis according to both patients and their referring clinician are listed in Table 1. No significant difference in participants' characteristics were observed between those with vs. without adult ADHD (Table 2), except for the number of ADHD criteria during childhood, which was higher among ADHD participants (Mann Whitney test, *p* = 0.02).

**Table 1:** Sample description.

	***N =* 17**
Age	37 (29–41)
**Gender**	
Women	7 (41%)
Men	10 (59%)
BMI	24 (22–24)
High school degree or more	7 (41%)
Unemployed	10 (59%)
Single	14 (82%)
Adult ADHD	13 (76%)
Combined	10 (83%)
Hyperactive/impulsive	1 (8%)
Inattentive	1 (8%)
**Main SUD at referral**	
Alcool	6 (35%)
Cannabis	4 (24%)
Cocaine	3 (18%)
Cathinones	2 (12%)
Benzodiazepines	1 (6%)
**Psychiatric comorbidity**	
Any mood disorder	7 (41%)
Any anxiety disorder	8 (47%)
Number of DSM5 disorders	4 (3–5)
Lifetime suicide attempt	8 (62%)

*Data are presented as median (interquartile range) or n (%). BMI, body mass index; ADHD, attention deficit hyperactivity disorder; SUD, substance use disorder*.

**Table 2:** Clinical and sociodemographic variables as a function of adult ADHD.

	**No adult ADHD**	**Adult ADHD**	***N***
	***N =* 4 (24%)**	***N =* 13 (76%)**	
Age	33 (29–39)	38 (36–41)	17
**Gender**			17
Women	1 (25%)	6 (46%)	
Men	3 (75%)	7 (54%)	
BMI	24 (22–24)	24 (22–24)	17
High school degree or more	1 (25%)	6 (46%)	17
WURS25 total score	54 (46–66)	64 (54–72)	15
ADHD criteria during childhood	5 (2–9)	8 (5–12)	17
ASRS-6 above cut off	3 (100%)	10 (83%)	
Unemployed	2 (50%)	8 (62%)	17
Single	4 (100%)	10 (77%)	17
**Current substance use**			
Current tobacco smoking	2 (50%)	8 (62%)	17
Current alcohol use	4 (100%)	11 (85%)	17
Current cannabis use	1 (25%)	7 (54%)	17
Current opioid use	1 (25%)	1 (8%)	17
Current cocaine use	0 (0%)	5 (38%)	16
**SUD diagnoses**			
Nicotine dependence	0 (0%)	5 (42%)	15
Any AUD	1 (25%)	5 (42%)	16
Any CUD	1 (25%)	8 (62%)	17
Any OUD	1 (25%)	2 (15%)	17
Any sedative use disorder	1 (25%)	2 (15%)	17
Any stimulant use disorder	0 (0%)	6 (46%)	17
Severity of DSM5 AUD			6
Early remission	0 (0%)	1 (20%)	
Severe	1 (100%)	4 (80%)	
Severity of DSM5 SUD			13
Mild to moderate	2 (100%)	5 (45%)	
Severe	0 (0%)	6 (55%)	
**Psychiatric comorbidity**			
Any mood disorder	2 (50%)	5 (38%)	17
Any anxiety disorder	2 (50%)	6 (46%)	17
Post-traumatic stress disorder	0 (0%)	3 (23%)	17
Total number of DSM5 diagnosis	3 (3–4)	5 (3–5)	17
Lifetime suicide attempt	2 (67%)	6 (60%)	13

*Data are presented as median (interquartile range) or n (%). SUDs measured by MINI DSM5, except for nicotine dependence, defined as FTND > 5. BMI, body mass index; ADHD, attention deficit hyperactivity disorder; WURS, Wender Utah Rating Scale 25 items; ASRS, Adult Self-Report Scale 6 items; AUD, alcohol use disorder; CUD, cannabis use disorder; OUD, opioid use disorder, SUD, substance use disorder; FTND, Fagerström Test for Nicotine Dependence*.

Thirteen (76%) participants were diagnosed with ADHD according to the DIVA-5. Seven patients (41%) presented with any comorbid mood disorder and eight (47%) with any comorbid anxiety disorder.

### ADHD (*n* = 13) vs. Non-ADHD (*n* = 4) Cases

#### Sociodemographic Data

ADHD cases seemed older and better-educated than non-ADHD cases, with a possibly higher proportion of women (46 vs. 25%). The distribution of marital and employment status seemed similar in both groups (Table 2).

Several interesting patterns appeared between ADHD and non-ADHD patients in Table 2. As regards sociodemographic data, gender ratio seem more balanced in the ADHD (46% women) vs. the non-ADHD group (25% women). ADHD cases seem younger that non-ADHD cases (33 vs. 38 years old) with higher level of education. As regards childhood ADHD symptoms, even non-ADHD participants had relatively high levels of WURS-25 and DIVA-5 scores, suggesting that they might have been diagnosed with ADHD if the assessments would have been conducted back then, but with probable remission in early adulthood. Most participants screened positive on the ASRS-6, including 100% no-ADHD cases. Conversely, two ADHD cases screened negative on the ASRS-6. When referring to the results of the DIVA-5 as a gold standard for ADHD diagnosis, the ASRS-6 showed 83% sensibility—same as for the WURS-25. Both screeners specificity were low (50% and lower), however they were deemed not interpretable due to the small sample size. As a whole, screening tools seemed to have a low diagnosis accuracy in the study sample, conversely to a previous study (37). Finally, as regards psychiatric comorbidity, the proportions of mood and anxiety disorders and lifetime suicide attempts were similar in both groups, noticing that all three PTSD cases also had ADHD.

#### Substance Use and SUDs

Both cocaine (38 vs. 0%) and cannabis use (54 vs. 25%) and their related disorders (46 vs. 0% and 62 vs. 25%, respectively) seemed more frequent in ADHD vs. non-ADHD participants (Table 2). Tobacco smoking was similar in both groups, however, 42% ADHD cases showed nicotine dependence compared to none in the non-ADHD group. Tobacco smoking was recorded by patients' response to the following question: “Do you currently smoke tobacco?,” and all current tobacco smokers fulfilled the Fagerström test for nicotine dependence (FTND).

**Figure 2:**
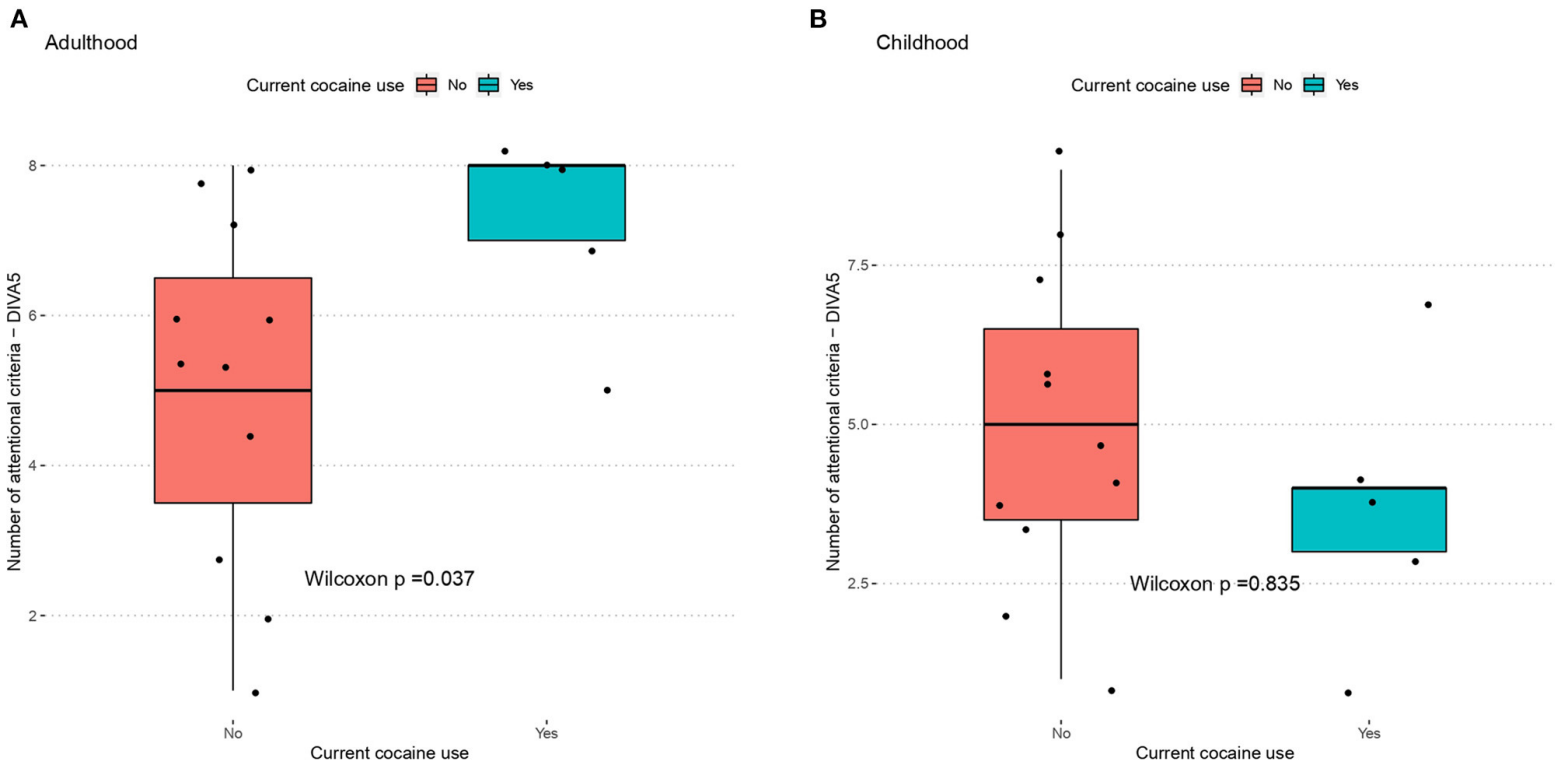
Cocaine use as a function of inattentive symptoms during **(A)** adulthood and **(B)** childhood. ADHD measured by the DIVA-5, cocaine use measured by anamnestic interview. BEARNI, Brief Evaluation for Alcohol Related Neuropsychological Impairment; FAB, Frontal Assessment Battery; DIVA, Diagnostic Interview for ADHD in Adult.

Although current cocaine use and lifetime Stimulant Use Disorder seemed strongly overrepresented in ADHD vs. non-ADHD cases, those differences were not significant. Thus, we further explored ADHD symptoms load as a function of these cocaine use patterns. By doing so, we evidenced that cocaine use was associated with increased ADHD symptoms, but only seen in adulthood, for inattention criteria (Mann whitney tests, *p* = 0.037, Cohen's *d* = 1.06 for cocaine use) (Figure 2).

#### Neurocognitive Measures

**Figure 3:**
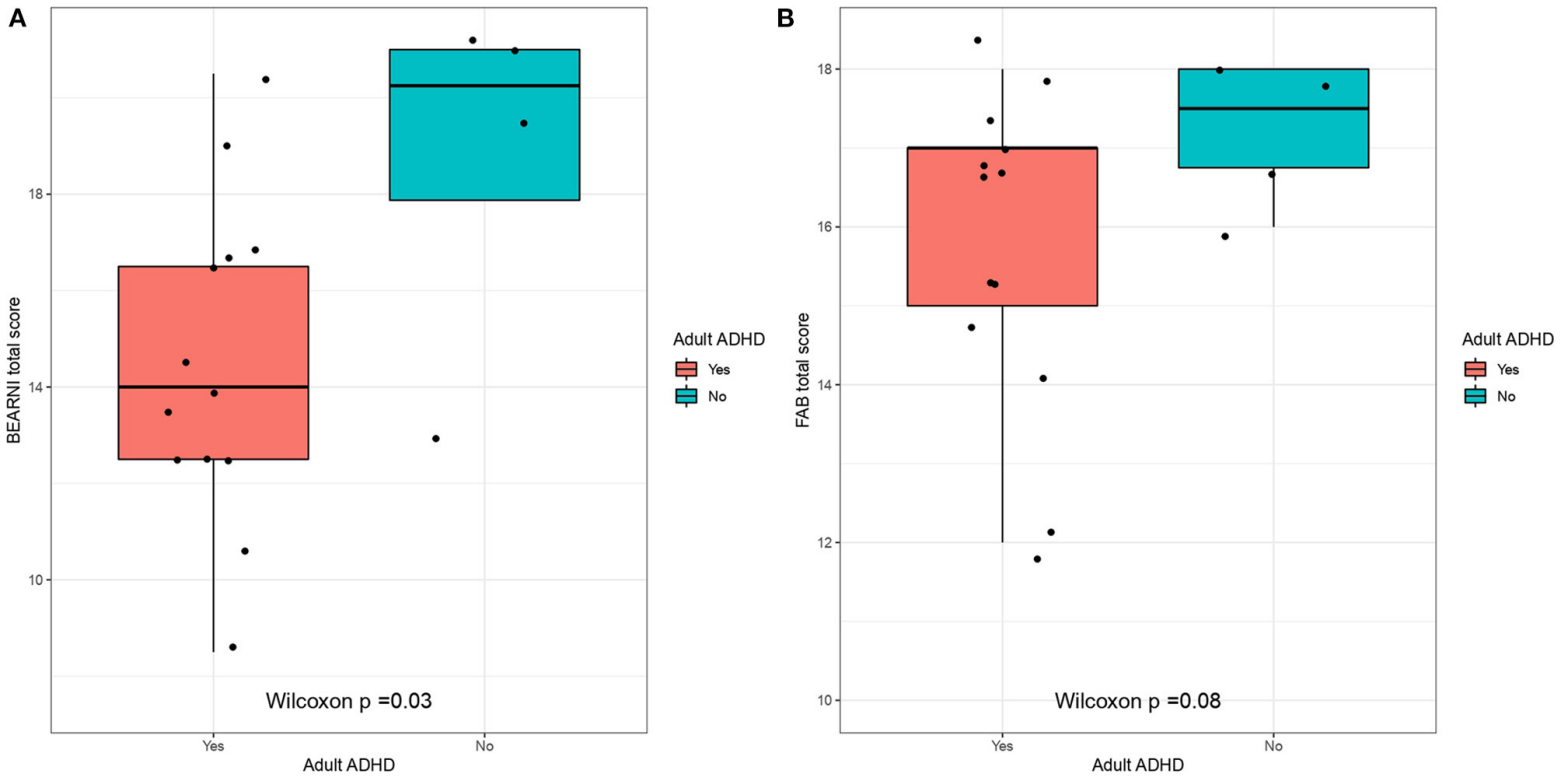
Neurocognitive scores as a function of ADHD. ADHD measured by the DIVA-5, neurocognitive profiles measured by **(A)** BEARNI and **(B)** FAB. BEARNI, Brief Evaluation for Alcohol Related Neuropsychological Impairment; FAB, Frontal Assessment Battery; DIVA, Diagnostic Interview for ADHD in Adults.

The BEARNI showed among the whole population a mean total score of 15.4 (*SD* = 3.8), which corresponded to moderate/severe impairment. ADHD participants (mean = 14.5; *SD* = 3.4) appeared significantly more altered than non-ADHD (18.6; *SD* = 3.8) on total score (Mann whitney test, *p* = 0.03; Cohen's *d* = 1.2) (Table 3). The FAB total score for the whole sample was 16 (*SD* = 2) which seems normal compared to the test norms and no significant difference was observed between the two groups. However, ADHD patients had lower scores on the Go-No Go subscale (mean = 2.2; *SD* = 0.9), compared to non-ADHD participants (mean = 3; *SD* = 0) but after the Holm's correction the difference was not significant (Mann whitney, uncorrected *p* = 0.025) (Figure 3). The WAIS-IV scores did not significantly differ from the norms and did not differ as a function of ADHD diagnosis.

**Table 3:** Neurocognitive measures as a function of adult ADHD.

	**No Adult ADHD**	**Adult ADHD**	***N***
	***N =* 4 (24%)**	***N =* 13 (76%)**	
BEARNI_TOTAL	20 (18–21)	14 (12–16)	17
FAB_TOTAL	18 (17–18)	17 (15–17)	17

*Data are presented as median (interquartile range). BEARNI, Brief Evaluation of Alcohol Related Neuropsychological Impairment; FAB, Frontal Assessment Battery*.

## Discussion

In this first case series of a sample of treatment-seeking SUD outpatients, who was thoroughly assessed for adult ADHD using a wide range of clinical and neurocognitive measures, participants did not report difficulties to attend and undergo all evaluations. Using exploratory analyses, we identified possible cognitive impairment associated with ADHD and relevant relationships between child vs. adult ADHD symptoms load and cocaine use—both warranting further exploration. We relied on a selected set of validated tools chosen to cover a wide range of symptoms and functioning domains.

### Feasibility

The extensive diagnosis strategy applied to SUD patients for diagnosing ADHD seemed extremely feasible. First, all patients attended and respected their schedule. This was not straightforward given the well-documented difficulty to plan and remind appointments for ADHD (38) and SUD people (39). In fact, both experiment executive difficulties with daily organization consequences, such as appointment attendance (40, 41). This was possibly supported by the text message and phone calls they received on the day before and by motivational bias, because of the entry procedure requiring the completion of several questionnaires before getting an appointment. Second, all participants finished the assessments. This finding was somehow unexpected because of the discomfort during lengthy activities of people with SUD, especially in case of comorbid ADHD. There are several suggestion to explain patients acceptance: (1) the procedure was presented in detail to participants beforehand; (2) they were helped for administrative formalities; (3) they benefited breaks between assessments and could ask for breaks at any time during the assessments; (4) evaluations were conducted by different clinicians; (5) lunch occurred onsite; (6) environment was convivial (coffee, healthcare staff availability); (7) patients had the same consultation room throughout the day (healthcare staff moved). Importantly as regards our global research aims, the DIVA-5 seemed to be well-accepted by participants, although it was the last evaluation of the day.

ADHD diagnosis among SUD adults already has been reported as feasible using the Conners' Adult ADHD Diagnostic Interview for DSM-IV (42), a thorough and demanding assessment. In line with this, our first case series also suggests the good feasibility of an even more extensive strategy to diagnose ADHD in this population. The large majority of participants tolerated the long and *a priori* tiring evaluation procedure well. During the final feedback interview, ADHD and non-ADHD patients reported moderate tiredness and argued that internal motivation to explore ADHD symptomatology helped them to support the procedure. We plan to incorporate proper satisfaction and feasibility measures in our assessments for the near future to assess theses subjective data using a more empirical method.

This preliminary study supports the feasibility of using the DIVA-5 as the core diagnostic instrument for ADHD among a full set of evaluations. However, a larger sample will be required in order to formally investigate its psychometric properties. Nonetheless, extensive strategy to diagnose ADHD in adults suffering from SUD seems relevant. A similarly extensive strategy has been used by Swedish researchers to diagnose participants in an interventional study (43), with no report of major refusal or attrition rates. However, this study did not precise if all assessments were done on a single day and its population strongly differed from ours regarding sociodemographic characteristics.

### Gender Balance

In our case series, the males:females ratio for ADHD was ~1 (46% women), thus possibly differing from the 1.5:1 usually reported (44). This may be explained by interactions between SUD, ADHD and gender, hypothesizing that, in SUD samples, gender balance would be reduced given that ADHD is a strong risk factor for SUD. SUDs are much more frequent for men (7.5%) compared to women (2.0%) (45) in the general population.

### Cocaine and Cannabis Use Patterns

ADHD participants were more likely to use cannabis and to suffer from cannabis use disorder than non-ADHD patients, and the same patterns were observed for cocaine. This may be due to the high score of sensation seeking (46) often reported in ADHD. This temperamental profile has been associated with multiple substance use experiments. These associations could also be related to the hypothesis of ADHD as a causal factor for lifetime cannabis use (47). Mirroring this, cannabis use could help ADHD patients to regulate their symptoms (as impulsivity, hyperactivity, anxiety, irritability), which is supported by patient's subjective motivation to use cannabis for its expected beneficial effects on ADHD symptoms (48). As regards cocaine, the self-medication hypothesis could be “classically” considered as an explanation. However, given the fact that the ADHD-cocaine association was only found for adult ADHD symptoms, but not for child, this finding may reflect the pharmacological effects of cocaine on individuals, who presented some childhood ADHD symptoms that increased after protracted cocaine use throughout their adulthood. This hypothesis of ADHD syndromes secondary to cocaine use—as is plausible for other mental disorders such as e.g., bipolar disorder (49)—has been suggested by our group, based on screening tools (50). It warrants further discussion and validation using structured interviews such as those conducted in the current study.

### Cognitive Profiles

On the whole sample, BEARNI total scores corresponded to moderate/severe impairments (<16), according to the test validation (51), with ADHD patients significantly more impaired than non-ADHD patients on the BEARNI total score. A recent study also found prominent neuropsychological impairments on executive functions in psychiatric adult outpatients seeking clinical evaluation of ADHD (52). There have been a large number of reports for cognitive function in ADHD. However, those reports are discrepant (53), owing to the various nature of the samples included in terms of sociodemographic and clinical profiles (54).

### Clinical Relevance of Assessment Procedures on ADHD Diagnosis and Treatment

The aim of this study was to describe patients' ability to undergo the full procedure and describe preliminary results on cognitive characteristics. Since both screening questionnaires and diagnostic interviews were used, we are able to report their initial diagnostic accuracy. ASRS-6 and WURS-25 sensibility was good when the scales were used separately or combined, however, they showed a non-acceptable false positive rate. The ASRS psychometric properties in SUD population have already been described, but remain inconsistent across studies. The false-positive rate appeared very high in one study (55), and acceptable but lower than sensitivity in others (13, 56). However, van de Glind et al. (13) identified a better specificity in participants for whom alcohol was the primary substance of abuse, compared to other substances (76 vs. 56%). This suggests an effect of substance type on ASRS specificity and could explain the poor ASRS specificity in our sample, where alcohol is the primary substance of abuse for only 35% patients. Other reasons might be at play, however, since other studies reported a higher specificity than sensitivity for the ASRS (86 vs. 61%) in adults seeking treatment for cannabis (57) or cocaine use disorders (16). Interestingly enough, in both studies the WURS specificity was lower than its sensitivity. Our sample size is too small yet to identify an effect of substance type on the psychometric qualities of screening assessments. Overall, it seems that the recommended ADHD screeners show inconstant, thus unsatisfactory properties, so that clinicians are encouraged to complete their evaluations when they strongly suspect ADHD, even when standardized screening was negative. Moreover, screening tools are especially expected to show very high sensitivity, at the possible cost of specificity. With that regards, the hyperactivity/ADHD subscale of the Strengths and Difficulties Questionnaire (SQQ) was recently validated in young adults and could represent an alternative to both the ASRS and the WURS (58). Thus, the authors found a high validity for the SQQ to distinguish ADHD and non-ADHD patients. However, further research is needed to explore its validity in SUD populations, especially with various primary substance of abuse.

Given the likely effect of substance type on the validity of screening questionnaires, one should bear in mind the crucial role of clinical interview and follow-up to diagnose adult ADHD in SUD populations. However, such a relatively unstructured approach seems more efficient when it is combined with standardized instruments. This may be explained by the fact that the clinical expression and impact of ADHD changes substantially over the lifespan. Thus, compared to childhood, adult ADHD is strongly represented by internalizing symptoms, impaired functioning and much higher comorbidity rates (59, 60). If screening questionnaires seem to not represent a sufficiently precise method, the DIVA-5 could be helpful, as it drives the clinician to investigate each DSM-5 ADHD criterion with additional clinical appreciation based on day life symptoms impact. The DIVA strongly highlights the needs to consider differential diagnosis and give the clinician a large freedom to do so. However, as regards our study, we deemed relevant to further use structured instruments to ascertain such diagnoses, for both clinical and research purposes. In this study we decided to use structured interviews to help the differential diagnosis process, but the interpretation of these interview results as a differential diagnosis or a comorbidity requested a clinical judgment. Globally, the DIVA-5 can be recommended as a useful help to diagnose ADHD among adults (with or without SUD) through the main steps of a diagnosis procedure (61), while leaving room for clinical investigations.

Finally, neuropsychological assessments are also often used to support the diagnosis procedure, as significant differences were identified between ADHD and non-ADHD on processing speed and work memory (20, 62). However, no significant difference was observed in our sample regarding processing speed and working memory between ADHD and non-ADHD. A more recent study also concluded to a limited utility of processing speed and working memory measures as indicators of the severity of ADHD (63). In fact, significant differences seem to disappear when IQ and depressive symptoms are included as covariate (21). Single neuropsychological measures seem to perform poorly in identifying ADHD, so that an extensive test battery may be necessary to control for the effects of comorbidity when searching for markers of ADHD diagnosis (64). These results could explain the non-significant difference observed in our study on WAIS-IV subtests, as it constitutes a single test measure performed with a highly comorbid sample.

One of the main aims of the evaluation procedure presented in the current manuscript was to provide therapeutic guidance to the clinician and explain it to the patient. This guidance included both pharmacological and non-pharmacological strategies, and the “hows and whens” of each proposed strategy. Although available evidence remains scarce, we relied on ADHD type (levels of inattention and hyperactivity), comorbidity profiles and functioning (both cognitive and daily life) to propose a personalized care plan to each participant, following the general recommendation for adult ADHD (26).

- As for stimulant medication, we recommended long-acting methylphenidate for five ADHD participants with strong functional impairment (combined and inattentive types) and atomoxetine in three participants. Atomoxetine was suggested because of age-associated risk factors of methylphenidate, potential comorbidity with bipolar disorder and current injection of psychostimulants (65, 66). For these participants, a delay before introducing methylphenidate was recommended (one after treating severe depressive symptoms, one after treating impulsiveness using valproic acid).
- Specific Cognitive and Behavioral Therapy (CBT) was systematically recommended in addition to pharmacological treatment in ADHD participants (26).
- The full procedure allowed to diagnose previously unidentified psychiatric comorbidities such as anxiety or mood disorders—especially PTSD and bipolar disorder; and cognitive impairment. Thus, in addition to recommendations on ADHD care and because of overlap between ADHD and comorbidities, we also suggested some interventions about these comorbidities. For instance, specific CBT for anxiety disorder was recommended for two ADHD participants and a specific exploration of bipolar disorder was suggested for one participant.
- Complete neurocognitive evaluation was recommended for two ADHD participants because of low scores in neurocognitive assessments (BEARNI, FAB, WAIS-IV subtests) and/or recent exacerbation of neurocognitive symptoms. Also, neurocognitive assessments led to recommend cognitive remediation for ADHD participants with strong executive difficulties.
- Finally, for non-ADHD participants, specific intervention targeting anxiety and mood disorders could be recommended.

The whole evaluation procedure resulted in personalized proposals for ADHD treatment, taking comorbidities into consideration as well as cognitive and emotional difficulties. Relevant psychological dimensions could also be identified in some cases, further increasing the personalization of both pharmacological or psychotherapeutic interventions. Also, the number of assessments facilitated the differential diagnosis to avoid false positive for ADHD. A major goal for treating burdensome mental conditions is functional recovery. We expect the personalized interventions proposed through our procedure to eventually lead to significant improvement of functional impairment, as was evidenced in the French expert centers for bipolar disorder, which use similarly thorough assessments as ours (67).

### Generalizability

We found a prevalence of ADHD of 76% among SUD outpatients. This frequency is considerably higher than others studies among SUD patients where ADHD is found for 15–25% of SUD patients (3, 8). It is difficult to date to compare these findings, noticing that our prevalence stands for people who were suspected for ADHD. Moreover, the study sample size was very small. For those reasons, the ADHD prevalence is not generalizable of all SUD patients.

### Limitations

The study has several limitations. First, the sample was very small, thereby reducing statistical power and results interpretation: a dimensional approach to describe ADHD symptoms intensity and evolution across a developmental spectrum would be interesting. Second, there might be a selection bias because of the entry procedure requirements. Patients had to complete several questionnaires to be evaluated and their clinicians had to complete a referral letter. As a result, maybe this procedure included only patients who did not had difficulties in assessments completion. Third, the procedure is fairly demanding in terms of resources. Moreover, there was no control group and no formal assessment of the tests scoring fidelity. Finally, we did not record age of onset of ADHD and SUD nor patients' background or developmental history which could have been helpful for a more comprehensive assessment of ADHD and SUD.

## Conclusion

We report here a detailed methodology for a reliable assessment of complex dual diagnoses such as ADHD, paving the way for a future validation study of major tools in the field. With a larger sample, we will be able to precisely describe the clinical and neurocognitive correlates of adult ADHD in severe SUD. Additionally, we will strive to identify the minimum set of assessments required for a reliable ADHD diagnosis in SUD populations, since not all clinicians or care settings will gather enough resources for using as many evaluations as we did.

These assessments are useful to refer patients to specific care settings for ADHD and SUD patients, that remain to be further developed, as specific Cognitive Behavioral Therapy for ADHD-SUD and specific neurocognitive interventions.

## Data Availability Statement

The raw data supporting the conclusions of this article will be made available by the authors, without undue reservation.

## Ethics Statement

The study was conducted according to the tenets of the Declaration of Helsinki (Declaration of Helsinki, 2013) and of Paris-Nanterre University ethics committee rules. It was formally approved by the relevant Ethics Committee (CPP sud-est IV, on February 22, 2021).

## Author Contributions

NT: conceptualization, methodology, investigation, formal analysis, and writing—original draft. LR: methodology, validation, writing—review and editing, and supervision. AM: investigation. AD: methodology, investigation, and resources. FV: resources and validation. EK: methodology, investigation, and writing—review and editing. RI: methodology, investigation, formal analysis, writing—review and editing, validation, and supervision. All authors contributed to the article and approved the submitted version.

## Funding

The positions of the clinicians who conducted the research are funded by Assistance Publique - Hôpitaux de Paris and Université Paris-Nanterre.

## Conflict of Interest

The authors declare that the research was conducted in the absence of any commercial or financial relationships that could be construed as a potential conflict of interest.

## Publisher's Note

All claims expressed in this article are solely those of the authors and do not necessarily represent those of their affiliated organizations, or those of the publisher, the editors and the reviewers. Any product that may be evaluated in this article, or claim that may be made by its manufacturer, is not guaranteed or endorsed by the publisher.
